# Neuroplasticity of the white matter tracts underlying recovery of diarrhea-predominant irritable bowel syndrome following acupuncture treatment

**DOI:** 10.3389/fnins.2024.1383041

**Published:** 2024-09-19

**Authors:** Jing Li, WingYi Ng, YongKang Liu, XiaoKun Fang, ZhongQiu Wang, LiXia Pei, XueHu Wei

**Affiliations:** ^1^Department of Radiology, Affiliated Hospital of Nanjing University of Chinese Medicine, Jiangsu Province Hospital of Chinese Medicine, Nanjing, China; ^2^Department of Acupuncture-Moxibustion and Rehabilitation, Affiliated Hospital of Nanjing University of Chinese Medicine, Jiangsu Province Hospital of Chinese Medicine, Nanjing, China; ^3^McLean Imaging Center, McLean Hospital, Harvard Medical School, Belmont, MA, United States; ^4^Department of Neurosurgery, Brigham and Women’s Hospital, Harvard Medical School, Boston, MA, United States

**Keywords:** irritable bowel syndrome, white matter fiber bundles, DTI, acupuncture, brain function

## Abstract

Irritable bowel syndrome (IBS) is a functional bowel disorder frequently associated with other pain syndromes and psychiatric conditions, including depression and anxiety. These abnormalities coincide with alterations in the brain’s structure, particularly in the thalamus and cingulate system. Acupuncture has been demonstrated to be highly effective in treating IBS. However, it remains unclear how white matter (WM) tracts change after acupuncture treatment, and whether the neuroplasticity of these tracts can serve as a neural marker to assist in the development of novel treatments. In this study, we aim to answer these questions by investigating longitudinal changes in the WM of the thalamus and cingulate system in a group of diarrhea-predominant irritable bowel syndrome (IBS-D) patients before and after acupuncture treatment. We found that after acupuncture treatment, as IBS symptoms improved, there were significant changes in the microstructure of the right thalamus radiation (TR) (*p* < 0.05) and the right cingulum hippocampus (CH) (*p* < 0.05). At the same time, patients with reduced IBS symptom severity scores (SSSs) were associated with the change of the right CH (*p* = 0.015, *r* = −0.491), while reduced depressive conditions correlated with the change of the left TR (*p* = 0.019, *r* = 0.418). In addition, the consequences for the quality of life (QOL) showed a correlation with the right cingulum [cingulate cortex (CC)] (*p* = 0.012, *r* = 0.504) and left TR (*p* = 0.027, *r* = −0.397). Our study highlighted the potential implications of neuroplasticity in WM tracts for IBS. Furthermore, these findings suggested that the right CH, TR, and right CC can serve as potential “biomarkers” of IBS-D recovery under acupuncture treatments.

## 1 Introduction

Irritable bowel syndrome (IBS) is a chronic condition characterized by dysfunction in the gastrointestinal tract (Pérez et al., 2008). Of all types, diarrhea-predominant irritable bowel syndrome (IBS-D) is the most common subtype (Lacy, 2016). IBS patients suffer from chronic abdominal pain or discomfort, stool irregularities, and bloating over an extended period (Longstreth et al., 2006; Weaver et al., 2016), and are also often comorbid with psychological disorders (e.g., depression or anxiety) or with comorbid insomnia (Tosic-Golubovic et al., 2010; Lacy, 2020; Hu et al., 2021). The interplay of biological and psychosocial disorders severely reduces the quality of life (QOL) in individuals with IBS (Whitehead et al., 1996; Zhu et al., 2015). To date, the pathogenesis of IBS remains unclear. Recent neurobiological studies have highlighted that IBS is currently viewed as a comprehensive disorder involving altered physical functions and disruptions in the gut–brain axis (Laskaratos et al., 2015; Mayer et al., 2015; Moloney et al., 2016). Long-term IBS led to alterations in the brain networks associated with viscerosensory and somatosensory information; pain processing; and the regulation of negative emotions such as anxiety and depression (Mayer et al., 2015; Moloney et al., 2016; Weaver et al., 2016; Mayer et al., 2023). Therefore, investigating changes in brain plasticity and their interactions can clarify their relation to IBS clinical features, providing new insights into the effectiveness and mechanism of treatments.

The examination of structural networks in IBS indicated that the thalamus and cingulate cortex (CC) play a critical role as network hubs in IBS (Labus et al., 2014), which is associated with various functional processes in IBS (Mayer et al., 2015; Weaver et al., 2016; Mayer et al., 2023). The thalamus is one of the key brain regions involved in receiving sensory input from the periphery (Mayer et al., 2015), regulating pain (May, 2008; Bhatt et al., 2019), and modulating negative moods such as anxiety and depression caused by chronic pain (Baliki and Apkarian, 2015; Brandl et al., 2022). The thalamus was commonly reported to be significantly affected by IBS (Laskaratos et al., 2015; Weaver et al., 2017; Mayer et al., 2023). Compared to healthy controls, IBS patients exhibit increased activation (Bouhassira et al., 2013) and alterations in brain gray matter networks in the thalamus (Labus et al., 2014). The CC is a key region of the pain matrix together thalamus response to pain perception and regulation in IBS (Apkarian et al., 2005; Baliki et al., 2006; Liu and Chen, 2009). Posterior CC is included in the default mode network and along with other brain regions within the default network (e.g., the hippocampus and parahippocampus) has been reported to be involved in anxiety modulation and stress inhibition (Sakamoto et al., 2005; Joëls, 2008; Ries et al., 2009; McLaren et al., 2016). Thus, IBS commonly shows changed gray matter volume (Davis et al., 2008) and decreased cortical thickness (Jiang et al., 2013) in CC.

In addition, significant changes in functional activity (Mayer et al., 2005; Bouhassira et al., 2013; Lowén et al., 2013) and disrupted functional connectivity between cingulate and other brain regions were widely observed associated with IBS (Lee et al., 2012; Hong et al., 2014; Weng et al., 2017). It has been commented that regions of the brain affected by IBS do not act in isolation but rather as an intricate network. Therefore, IBS is widely associated with alterations in the white matter (WM) framework that modulates and transmits signals in the brain (Chen et al., 2011; Ellingson et al., 2013;Fang et al., 2017; Hubbard et al., 2018; Witt et al., 2019). The cingulate bundle forms a nearly complete loop, extending from the orbitofrontal cortex, along the corpus callosum, and connecting to the parahippocampus. This bundle is found to be engaged in various processes, including pain, depression, and post-traumatic stress disorder (Bubb et al., 2018; Weis et al., 2018). The cingulate was found with a significant change in the diffusion property in IBS patients (Chen et al., 2011; Hubbard et al., 2018). At the same time, researchers found that IBS patients, compared to healthy individuals, exhibit significant alterations in the WM microstructure of the bilateral thalamus and primary sensory cortex (Chen et al., 2011; Ellingson et al., 2013). Some current studies have confirmed that long-term suffering from IBS results in WM microstructural changes within the brain (Fang et al., 2017; Hubbard et al., 2018; Witt et al., 2019). There difference in the WM plasticity in IBS suggests that assessing the effect of microstructural WM integrity by IBS may unravel more reasons behind the IBS’s behavior disorder.

Diffusion magnetic resonance imaging (MRI) can be used to evaluate the WM structural plasticity changes non-invasively with precise assessment at the level of individual voxels of both the magnitude and directionality of water diffusion (Jezzard et al., 1994). The most widely used diffusion tensor imaging (DTI) metrics are fractional anisotropy (FA), radial diffusivity (RD), axial diffusivity (AD), and mean diffusivity (MD). FA, which ranges between 0 (isotropic diffusion) and 1 (unidirectional diffusion), indicates the extent to which diffusion is directionally dependent. It provides information about the orientation, density, and coherence of fibers within a voxel. RD indicates the diffusion properties perpendicular to the direction of WM fibers, whereas AD describes the microscopic characteristics parallel to axonal tracts. MD is a measure of the overall magnitude of water diffusion in a voxel. It is influenced by the density of physical barriers that impede water movement.

Clinically, many methods of treating IBS have been proposed (Bijkerk et al., 2004). Acupuncture, as a traditional Chinese medicine treatment method that offers fewer side effects and cost-effective alternative, has been widely used in the treatment of functional gastrointestinal diseases (Lan et al., 2014). In addition, acupuncture has been proven to effectively mitigate symptoms of IBS including abdominal pain, distention, obstructed defecation, depression, and anxiety (Wu et al., 2019; Pei et al., 2020). So far, the central mechanisms of acupuncture for IBS remain unclear. We found some related functional MRI studies (Ma et al., 2020; Geng et al., 2021; Zhao et al., 2021) while there was still a lack of structural MRI studies—especially WM tract—specializing in this field.

The current study was conducted with IBS-D patients and a longitudinal intervention design was used before and after a 6-week acupuncture treatment. We also tracked the improvements in IBS symptoms and accompanying changes in the WM fibers involving the thalamus and cingulate gyrus regions that are thought to carry signals critical to IBS (Ellingson et al., 2013; Mayer et al., 2015; Weaver et al., 2016). These fibers included the thalamus radiation (TR) tract, cingulum cingulate, and cingulum hippocampus. On the basis that acupuncture could relieve the symptoms of IBS-D and the three WM mentioned above are closely associated with IBS-D, we hypothesized that the neuroplastic change of these WM tracts would contribute to the progression and recovery of the IBS condition after acupuncture treatment. This research could provide valuable insights into the neural mechanisms underlying recovery, potentially leading to more effective treatment strategies for IBS-D patients.

## 2 Materials and methods

### 2.1 Participants

We consecutively recruited 39 right-handed IBS patients (28 men/11 women, mean age 41.18 ± 10.43 years) from the Affiliated Hospital of Nanjing University of Chinese Medicine, Nanjing, China. Subjects recruited for our study underwent evaluation based on a series of criteria set by gastroenterologists who were experienced in diagnosing functional bowel diseases and excluding organic diseases. The inclusion criteria were as follows: (1) subjects that meet the Rome III criteria (Drossman, 2006) for IBS of diarrhea type (IBS-D); (2) age between 18 and 55 years; (3) disease course over 6 months; (4) IBS symptom severity score (IBS–SSS) is above 75 on baseline; and (5) no drug treatment within 2 weeks and no history of acupuncture treatment within 3 months regarding IBS. Exclusion criteria were as follows: (1) organic gastrointestinal disease or history of gastrointestinal surgery; (2) current or past psychiatric illness or substance abuse; (3) any major medical or neurological conditions; (4) medication such as selective serotonin reuptake inhibitors, aspirin, non-steroidal anti-inflammatory drugs that affect gastrointestinal motility for more than 2 weeks before enrollment; and (5) metal sensitivity or afraid of the needle.

All IBS-D patients received about 6 weeks of acupuncture treatment. Clinical and psychometric measurements, as well as the high-angular and spatial resolution diffusion MRI data, were acquired from each participant when they came to the hospital for diagnosis and the following day when they completed the acupuncture stimulations for 6 consecutive weeks mentioned in the following acupuncture protocol. This prospective study protocol was approved by the local Medical Research Ethics Committee (reference number: 2016NL-078-03). Written informed consent was obtained from all participants prior to the study.

### 2.2 Acupuncture protocol

Acupuncture can unblock the meridians and collaterals, regulate the function of qi and blood, support health, and expel pathogens by stimulating acupoints (Chao and Zhang, 2014). It was previously reported that acupuncture and moxibustion are effective for IBS patients (Pei et al., 2020). The acupoints were identified based on the method of point location issued by the World Health Organization (WHO), including Bai Hui (DU20), Yin Tang (EX-HN3), bilateral Tai Chong (LR3), Zu Sanli (ST36), San Yinjiao (SP6), Tian Shu (ST25), and Shang Juxu (ST37). Acupuncture treatments were conducted after skin disinfection using sterile acupuncture needles (0.30 mm in diameter, 40 mm long, Hwatuo, Suzhou Medical Appliance Manufactory, Jiangsu, China) in accordance with the standard permissible depth of insertion for each acupoint. DU20 and EX-HN3 were punctured obliquely 10 and 5 mm into the skin, respectively. ST36, ST37, and SP6 were punctured 25 mm into the skin, while ST25 and LR3 were punctured 25–40 and 15 mm, respectively. Specialized acupuncturists with more than 6 years of clinical experience were responsible for achieving an optimal acupuncture response, known as Deqi, characterized by sensations of soreness, numbness, heaviness, and distention, through the interventions of twirling, lifting, and thrusting at the specified seven acupoints. The duration of the therapy was long for 30 min while a 10-s stimulation of needles was manipulated twice every 10 min. Acupuncture treatment was administered once daily, up to three times a week, for a total duration of 6 weeks.

### 2.3 Clinical and psychometric measures

Primary clinical severity measures included disease duration (duration of IBS symptoms in years), IBS–SSS (Francis et al., 1997), and IBS–QOL (Patrick et al., 1998). The Hamilton Anxiety Scale (HAMA) (Hamilton, 1960) was applied to assess the degree of anxiety. All outcomes were evaluated before and after acupuncture therapy.

### 2.4 MRI data acquisition

Scanning of the dataset was performed on a 3 T MRI scanner (Siemens). The DTI data were acquired with isotropic 2.0 mm^3^ spatial resolution and full brain coverage. Other imaging parameters were as follows: matrix size = 128 × 128, repetition time (TR) = 95 ms, echo time (TE) = 10,500 ms, and 74 slices with no interslice gap. Each DTI session consisted of 30 diffusion directions (*b* = 1,000 s/mm^2^) and 2 images without diffusion weighting (*b* = 0 s/mm^2^). For each participant, a high-resolution structural T1 with a 1-mm resolution of the whole brain was acquired. The T1-weighted image parameters were as follows: matrix = 224 × 256; TR = 2,300 s; and TE = 2.19 ms.

### 2.5 Imaging data preprocessing

The DTI data was preprocessed via functional magnetic resonance imaging of the brain (FMRIB) software library (FSL)^1^. First, image distortions and subject head motion were corrected using FMRIB’s diffusion toolbox (FDT). Then, non-brain tissue was removed via a brain extraction tool (BET). Finally, individual FA maps and S0 maps (raw T2 signal with no diffusion weighting) were locally fitted.

### 2.6 Automatic fiber tracking

WM fiber tracking and tract quantification were performed using the automated fiber quantification (AFQ) software.^2^ First, we used mrDiffusion toolbox^3^ to finish a converting preprocessed step to create a dt6.mat file. This preprocessing included motion correction of the diffusion-weighted (DW) MRI images, coregistration of the DW MRI images to the T1, realignment of the anterior commissure–posterior commissure line, and tensor calculation. Then, the fiber tracking and tract quantification process followed the processing steps described by Yeatman et al. (2012). The tractography step using the deterministic streamlines tracking algorithm was initiated from each WM voxel with an FA > 0.3. Individual streamline integration was terminated based on two standard criteria: tracking was halted if the FA estimated at the current position was <0.2 and if the minimum angle between the last path segment and the next step direction exceeded 45°. Finally, selected tracts were sampled into 100 evenly spaced nodes, spanning termination points at the gray–WM boundary, and then FA, MD, AD, and RD of each node were mapped onto each tract. These diffusion parameters were used as the primary estimation parameters for further analysis.

### 2.7 Statistical analysis

In our study, clinical and psychometric measures of a total of 39 patients were included in the behavioral statistical analysis. For the image data assessment, after excluding the missing scanning and head motion, there were 31 IBS-D patients (21 men/10 women with a mean age of 40.774 ± 10.35 years) were included in the further brain data analysis. The group was created with the sample size to be large enough to detect tract-based differences in WM microstructure (Chen et al., 2011; Ellingson et al., 2013; Fang et al., 2017; Hubbard et al., 2018; Witt et al., 2019; Gronemann et al., 2020).

We started with the statistical analysis of the clinical and psychometric measures. Before treatment, Pearson’s correlation coefficients were first calculated to investigate the relationship between clinical and psychometric measures in IBS-D patients. This test involved IBS-SSS, QOL, HAMA, age, gender, education, and disease duration. Then, before and after the treatment, a paired *t*-test was used separately to estimate the changes in IBS symptom indicators in IBS-SSS, QOL, and HAMA to measure whether acupuncture treatment is effective in improving the symptoms of IBS-D patients.

Following the behavior test, the evaluation of the change of target fiber tracts between before and after treatment was performed in this step. During the automatic tracing process, we segmented each fiber tract as 100 nodes and calculated the FA, MD, RD, and AD parameters for each node. Based on this, we averaged each parameter across 100 nodes of each tract to create a single estimated value of each diffusion property (mean_ FA, mean_ MD, mean_ RD, and mean_ AD) for each participant; each averaged parameter value was then compared by paired *t*-test to assess change over the time of treatment (before and after treatment). Subsequently, the same paired *t*–test analysis was also used to test the change of each diffusion property at each node (100 nodes in total) of each selected tract, which investigated the neuroplastic change of point-wise levels along the tract trajectory.

We further examined the association between the change of available clinical measurements and WM diffusion properties by correlation analysis. Correlations were considered very weak for ± (0 ≤ *r* ≤ 0.2), weak for ± (0.2 ≤ *r* ≤ 0.4), moderate for ± (0.4 ≤ *r* ≤ 0.6), strong for ± (0.6 ≤ *r* ≤ 0.8), and very strong for ± (0.8 ≤ *r* ≤ 1.0).

For all *t*-tests, the normal distribution of the dataset of each parameter has to be to select the correct statistical analysis method. In our study, the threshold at a level of uncorrected *p* < 0.05 of statistical parametric maps was considered. For multiple comparison corrections, we followed previous studies with the AFQ method (García-Pentón et al., 2016; Li et al., 2020). The corrected significant threshold for mean diffusion metrics and point-wise univariate statistics of each tract was *p* < 0.05 with a false discovery rate (FDR) (Benjamini and Hochberg, 1995).

## 3 Results

### 3.1 Correlation between clinical and psychometric measures

**Figure 1:**
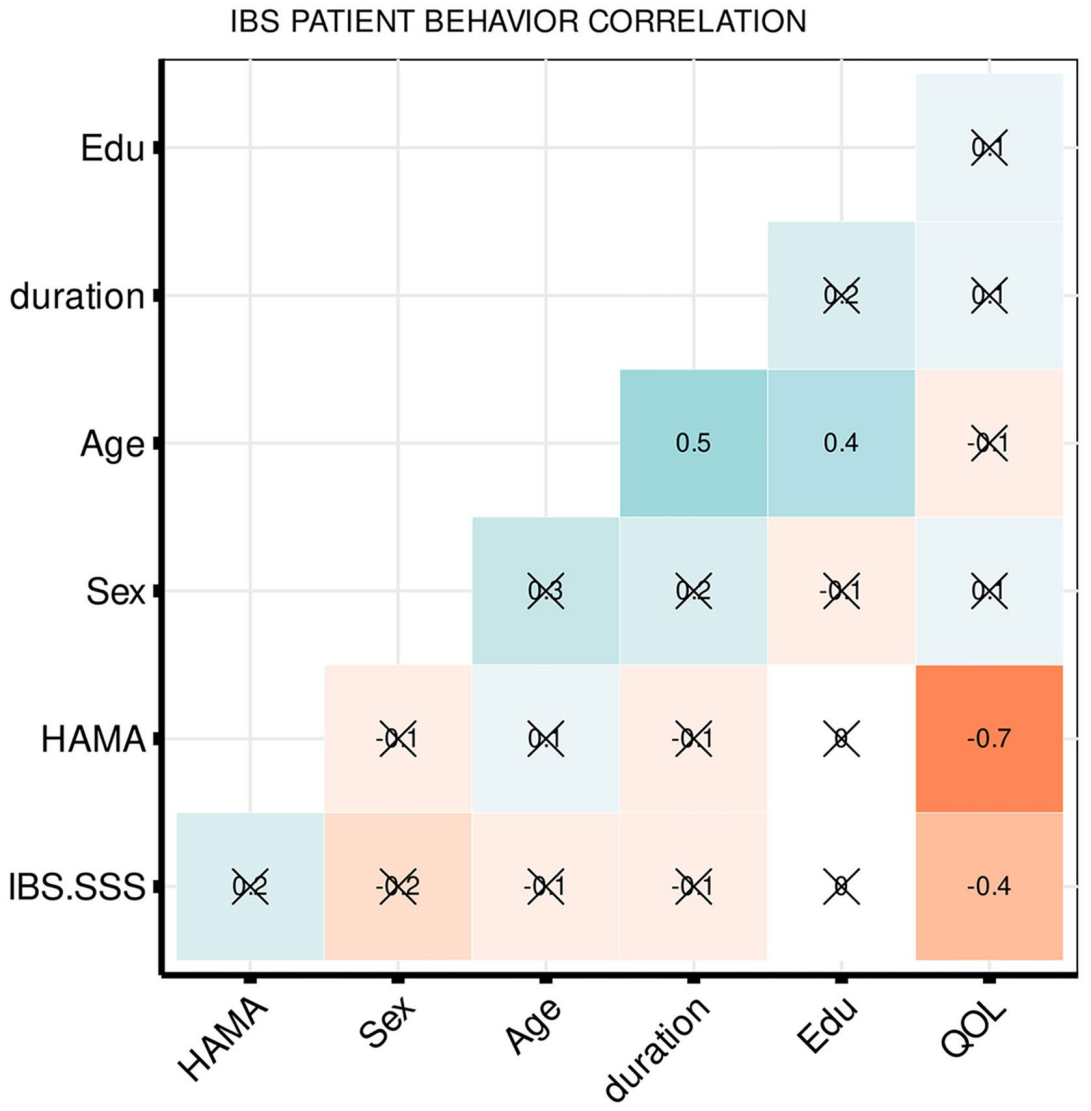
Heatmap summarizing pearson correlation coefficients (*R*) for clinical and psychometric measures. The values in the matrix represent the correlation between the corresponding measurements on the *x*-axis and *y*-axis. The weak and very weak correlations between the *x*-axis and *y*-axis variables were flagged with “x” (*R* < 0.4).

The partial correlation coefficients demonstrated that the IBS–SSS significantly negatively correlated with IBS–QOL (*r* = −0.4355, *p* = 0.01) and IBS–QOL significantly negatively correlated with HAMA (*r* = −0.7213, *p* = 1.49E−06), as shown in Figure 1.

### 3.2 Improvement of clinical and psychometric status in IBS-D patients after acupuncture treatment

**Figure 2:**
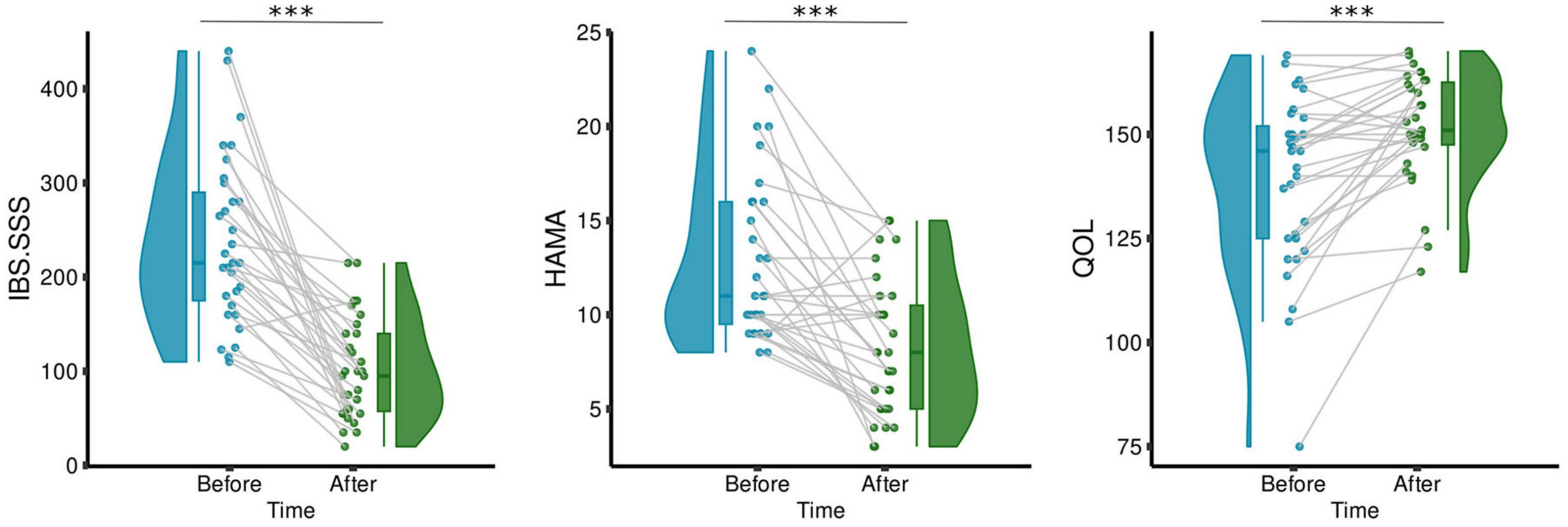
Change of clinical, psychometric scores after treatment. The boxplots show the median, quartiles, 1.5 * interquartile range, and all individual data points. The range in the violin plot shows the distribution from the minimum value to the maximum clinical or psychometric scores. The height represents the maximum value, and the width represents the number of subjects distributed at the corresponding value, ***p < < 0.0001.

To investigate whether acupuncture treatment improves the symptoms, we performed paired *t*-tests between the measurements taken before and after the 6-week acupuncture treatment for IBS–SSS, QOL, and HAMA. The results demonstrated that compared with before treatment, IBS–SSS (*t* = −7.8, *p* = 1.06E−08) and HAMA (*t* = −5.3, *p* = 1.11E−05) decreased significantly, while QOL increased significantly (*t* = 4.7, *p* = 5.55E−05) (see Figure 2).

### 3.3 Change of WM tract microstructure of IBS-D patients after acupuncture treatment

**Figure 3:**
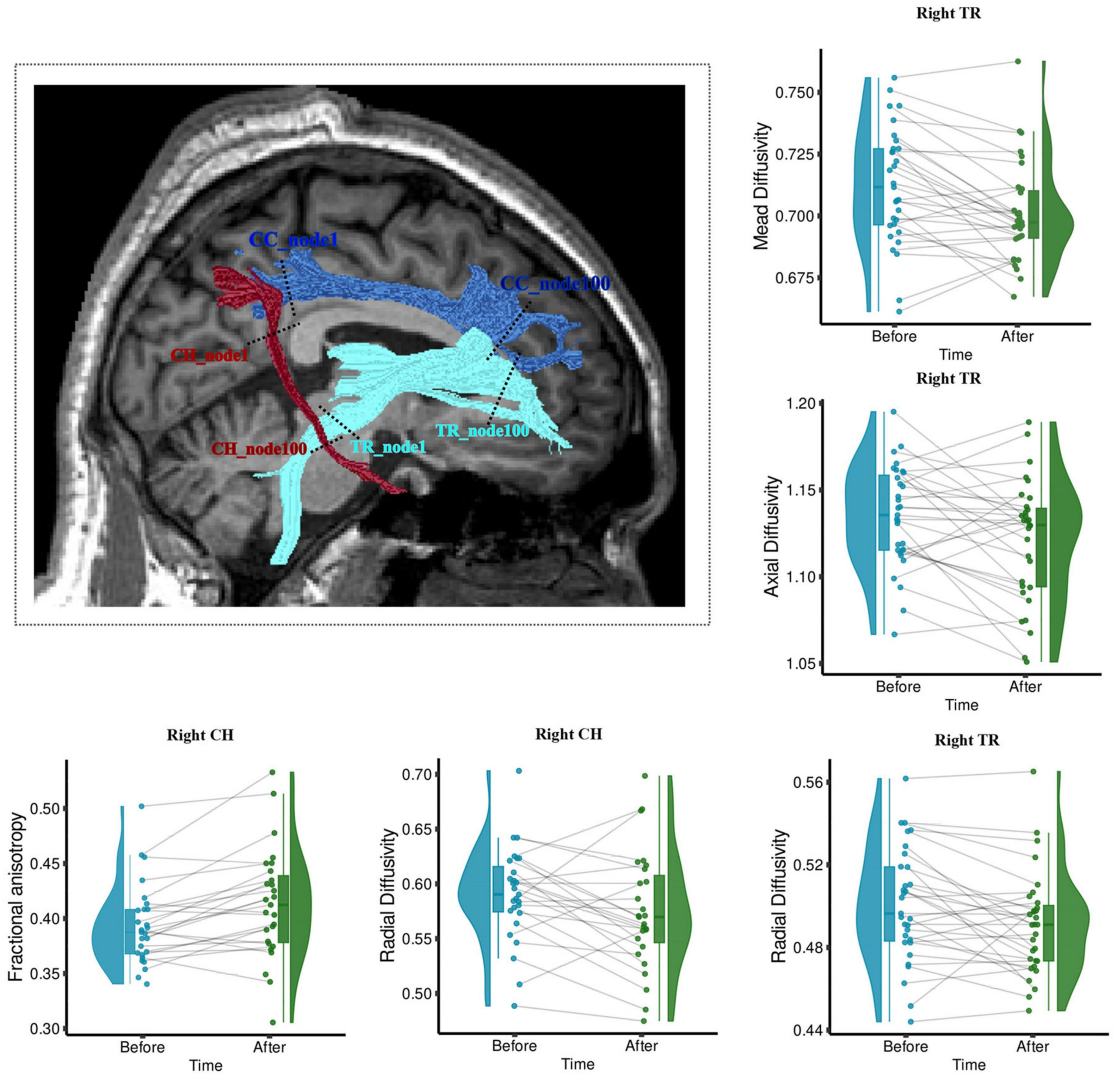
Wm fiber bundle with longitudinally changed diffusion properties after acupuncture treatment. The brain figure shows the location of the right Tr and Ch. Right Ch with increased Fa and decreased Rd; Right Tr with decreased Md, Ad, and Rd. The boxplots show the longitudinal changes in the diffusion parameters of fiber tracts before and after treatment, with the median, quartiles, 1.5 * interquartile range, and all individual data points. Fa, fractional anisotropy; Md, mean diffusivity; Ad, axial diffusivity; Rd, radial diffusivity. The range in the violin plot shows the distribution from the minimum value to the maximum diffusion parameters of fiber tracts. **p < 0.05, Fdr Corrected; *p < 0.05.

The longitudinal measurement of WM structure shows that after the 6-week acupuncture treatment, there was a significant change in the WM tract metrics (FA, MD, AD, and RD) of the right TR and right cingulum hippocampus (CH). Right TR significantly decreased in MD (*t* = −3.6, *p* < 0.05 FDR corrected), AD (*t* = −2.62, *p* = 0.013), and RD (*t* = −2.6, *p* = 0.014), as shown in Figure 3. Right CH showed a significantly increased mean FA (*t* = 3.78, *p* < 0.05 FDR corrected) and decreased RD (*t* = −2.2, *p* < 0.04), as shown in Figure 3.

### 3.4 Difference of WM in point-wise levels after acupuncture treatment

**Figure 4:**
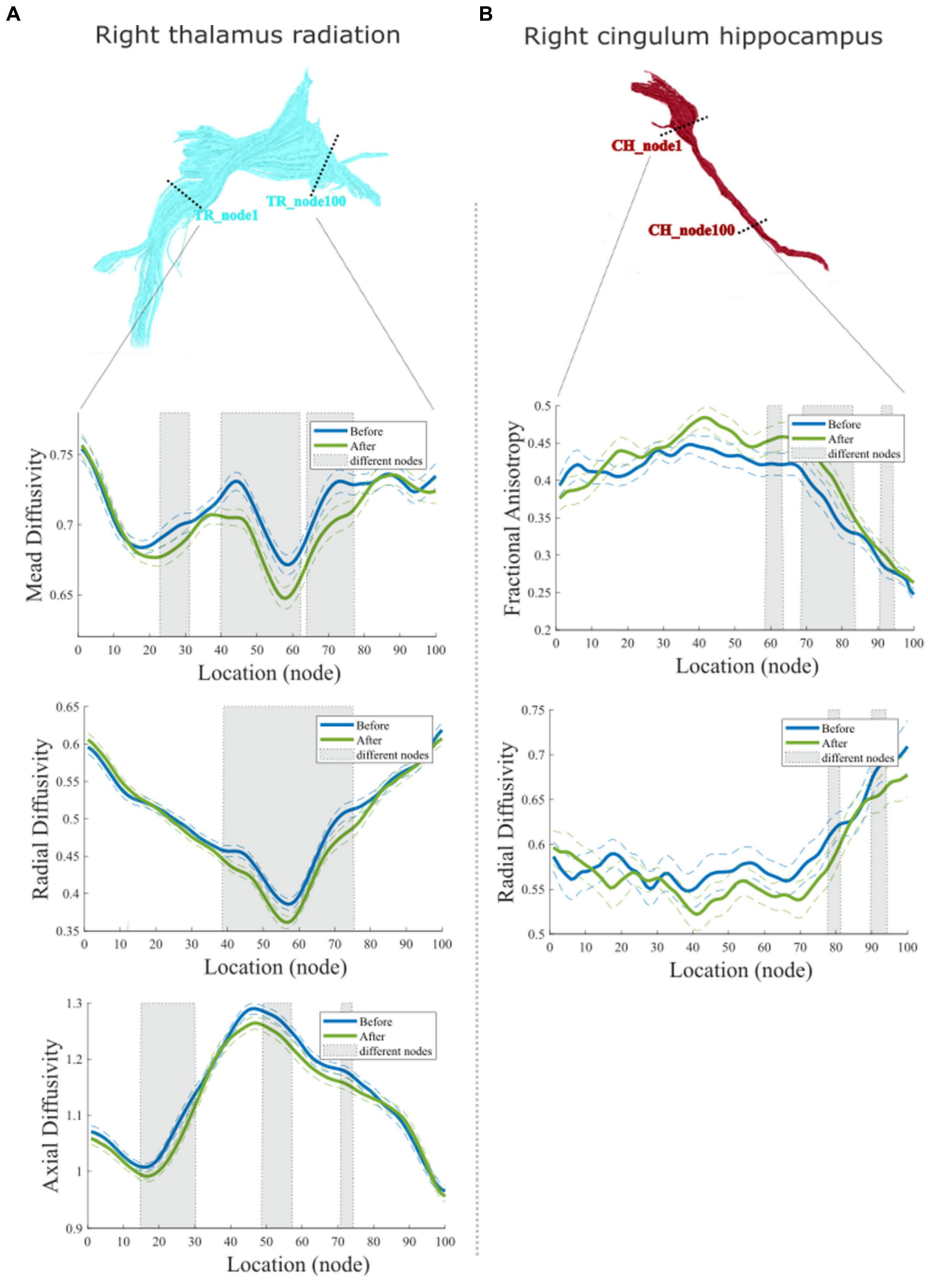
Point-wise comparisons for diffusion characteristics representing 100 equidistant nodes (segment) along the tract trajectory before and after acupuncture treatment. **(A)** Comparison of right Tr; **(B)** Comparison of right Ch. In each box plot, the blue solid line refers to the mean diffusion value of the tract before treatment, while the green solid line refers to the mean diffusion value of the tract before and after treatment. The dashed lines denote the standard deviation (Sd) values. Shaded areas represent nodes along the tract with significant statistical differences (*p* < 0.05, Fdr Corrected).

Furthermore, accurate point-wise comparisons were acquired to investigate the change along the tract trajectory between before and after the 6-week acupuncture treatment. As seen in Figure 4A, for right TR, the MD values decreased (*p* < 0.05) mainly in the locations of nodes 23–31, 40–62, and 65–77. RD values of right TR were mainly reduced in the middle sections (nodes 39–75), while AD decreased in three smaller clusters (nodes 14–30, 48–57, and 70–74, *p* < 0.05). The point-wise levels test in right CH show that after acupuncture treatment, the right CH regional locations with increased FA (nodes 59–63, 69–83, and 90–94, *p* < 0.05) and decreased RD (nodes 77–81, 90–94, *p* < 0.05) primary located in the nodes relatively close to right parahippocampus (see Figure 4B).

### 3.5 Correlation between symptom improvement and the change of WM tract microstructure

**Figure 5:**
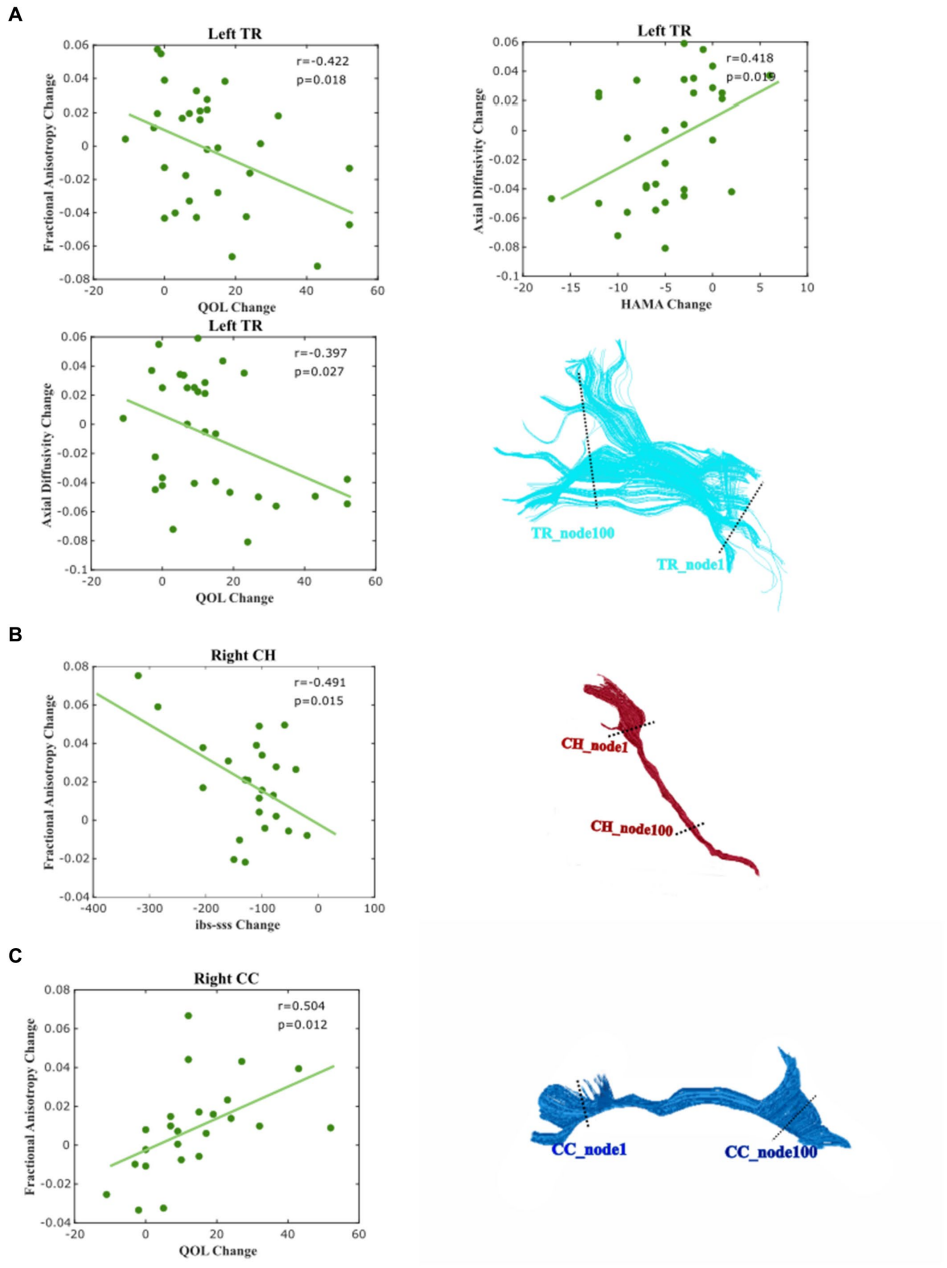
Correlation between the changed diffusion feature and clinical and psychological score. Correlations were considered very weak for ± (0 ≤ *R* ≤ 0.2), weak for ± (0.2 ≤ *R* ≤ 0.4), moderate for ± (0.4 ≤ *R* ≤ 0.6), strong for ± (0.6 ≤ *R* ≤ 0.8), and very strong for ± (0.8 ≤ *R* ≤ 1.0). **(A)** Left Tr, **(B)** Right Ch, and **(C)** Right Cingulum (CC).

Finally, we examined the relative WM changes in relation to the recovery of IBS by the correlation analysis between the changed IBS symptoms and the change of mean diffusion properties of each tract (see Figure 5). We found that the change in the IBS–SSS score is moderately related to plasticity change in the right CH and shows a negative correlation in FA (*r* = −0.491, *p* = 0.015). The improvement of QOL correlated with the mean FA of left TR (*r* = −0.422, *p* = 0.0198) weakly, and right CC (*r* = −0.504, *p* = 0.012) moderately. For the measurement of AD, we found that the change of mean AD of left TR was moderately correlated with the improvement of HAMA (*r* = 0.418, *p* = 0.019) and QOL (*r* = −0.397, *p* = 0.027).

## 4 Discussion

The present longitudinal study evaluated disease-related neuroplastic diffusion characteristic alterations in WM connectivity in individuals with IBS-D before and after the 6-week acupuncture stimulation. We observed a significant change in the WM diffusional characteristics in the right thalamic radiation (TR), right CH tract, and along with significant improvements in clinical and psychometric symptoms of IBS-D after acupuncture treatment. In the same line, the improvement of IBS–SSS, QOL, and HAMA were found to be associated with altered right CH, left TR, and right cingulum (CC). This study provided evidence that acupuncture treatment significantly improved the IBS symptoms. Importantly, the present longitudinal study demonstrated the neuroplasticity of the WM structural network, involving the thalamus and cingulum cingulate, which are network hubs critical for information flow in IBS-D, underlying the recovery of IBS-D under acupuncture treatment.

The correlation between the clinical and physiological score of IBS-D suggested that IBS-SSS was significantly correlated with QOL, which is consistent with the previous study that people suffering from chronic functional bowel disorders might be accompanied by reductive QOL (Gralnek et al., 2000; Halder et al., 2004; Singh et al., 2015). Patients with IBS often experience emotional issues, including symptoms of depression and anxiety, along with expressing neurotic personality characteristics (Pérez et al., 2008; Tosic-Golubovic et al., 2010). A significant correlation between the HAMA and QOL indicated that long-term negative emotions reduced the QOL in IBS patients (Zhu et al., 2015).

Our results showed that, after the 6-week acupuncture treatment IBS–SSS significantly decreased. This finding was consistent with multiple previous clinical trial studies that acupuncture treatment improved outcomes for people with IBS (Huang, 2009; Ruepert et al., 2011; Li et al., 2012). In addition, individuals’ IBS symptoms had improved, and they had experienced a reduction in their HAMA score along with an improved QOL. This suggested that their treatment or intervention had a positive impact on their overall wellbeing.

Previous research has shown that patients with IBS, who exhibited widespread network changes, most commonly displayed microstructural differences in the thalamus and cingulate gyrus compared to healthy controls (Weaver et al., 2016; Mayer et al., 2023). Evidence of significant microstructural changes indicated that IBS patients differ from healthy controls in the WM diffusion characteristics of FA, AD, RD, and MD in the thalamus and cingulate gyrus-related network (Chen et al., 2011; Ellingson et al., 2013; Zhou et al., 2013; Fang et al., 2017; Hubbard et al., 2018). Changed FA and MD is a highly reflected alteration of WM integrity, high FA and low MD are thought to indicate high WM integrity (Alexander et al., 2007). In turn, our study revealed that thalamic radiate tracts, cingulate hippocampal tracts, and cingulate tracts underwent plastic neural changes after acupuncture treatment, and these changes were associated with symptom recovery in IBS. Specifically, the microstructural changes, as well as the most prominent findings that are significantly associated with improvement in IBS symptoms, were observed in WM tracts involving the right thalamus and cingulate gyrus. This finding might be related to the highlights that the right cingulate gyrus and right thalamus are critical for information flow in IBS (Labus et al., 2014). Besides, the major underlying cause of abdominal discomfort symptoms, such as stomach pain and bloating, in people with IBS-D is the intricate process of visceral hypersensitivity, including hyperalgesia and hypervigilance. The human brain exhibits a distinct allocation of tasks and processing of information between its left and right hemispheres, with the right hemisphere being related to intuitive perception. The results of lateralization among the right hemisphere were also shown among IBS patients as compared to normal subjects (Fang et al., 2017) suggesting the predominantly affected region of IBS. Besides, similar lateralization after acupuncture was shown in our findings (Ma et al., 2020). We believe that acupuncture may stimulate more areas of the right hemisphere to relieve visceral hypersensitivity and emotional processing.

We found that accompanied by the change of functional symptoms, the right TR that connected the thalamus to the prefrontal cortex showed significantly decreased MD, AD, and RD after the 6-week acupuncture therapy. The thalamus plays an important role in both filtering sensory information and emotional regulation (Herrero et al., 2002). Previous studies of IBS have reported that the thalamus and prefrontal cortex are key regions in the human brain responsible for sensory sensitivity and pain modulatory/analgesic effects (Mayer et al., 2015, 2023). Patients with IBS show lower FA and higher MD in the thalamus and a higher number of tracts connecting the thalamus to the prefrontal cortex (Ellingson et al., 2013). Furthermore, alterations in the diffusion property parts of the anterior right TR were dementated and correlated with symptom severity of chronic pelvic pain (Farmer et al., 2015; Huang et al., 2016; Sean et al., 2023). Extrapolating this, reduced pain signaling may lead to reduced central sensitization of spinothalamic afferents after the treatment, so that, WM tracts associated with pain also undergo significant neuroplastic changes over time.

On the left side, changed diffusion characteristics of TR (were significantly correlated with alteration of QOL and HAMA). Left TR in particular in the anterior was reported as more likely to be related to negative feelings such as sadness (Coenen et al., 2012). Altered disruption of structural network centrality of the left thalamus is correlated with psychological symptoms and disease duration in patients with longstanding gut inflammation (Turkiewicz et al., 2021). Results from the current study provide new information that neuroplasticity change of left TR is therefore critical for emotional regulation in IBS-D patients during recovery. In addition, our effects may imply that the left TR and right TR may play different roles in longstanding gut diseases.

In addition to WM effects in the thalamic radiation, we found significant changes in right CH with increased FA and RD. This fiber tract extends from the posterior cingulate gyrus (PCC) and connects to the parahippocampal areas. The PCC and its connections to other brain regions serve as an important component of the default mode network (DMN), which is implicated in perceptual aspects of pain, for instance, pain attention (Kucyi and Davis, 2015). In addition, the study of IBS treatment highlights that DMN including posterior cingulate serves as a potential marker of treatment effects for chronic pain in IBS (Letzen et al., 2013). Although parahippocampal region is not typically associated directly with the sensation of pain, there has been mention of the parahippocampal gyrus being activated in certain types of pain or pain-related memory about the contextual and emotional aspects of pain (Smallwood et al., 2013). The right parahippocampal is one of the structural network hubs in IBS (Labus et al., 2014) and is significantly altered in volume in patients with chronic pain (Smallwood et al., 2013). The present study found that right CH as a structural connectivity between PCC and parahippocampal gyrus showed significant change after treatment. Furthermore, we found that the changed diffusion feature of the right CH significantly correlated with the changed IBS–SSS. Based on the existing evidence that DMN interaction with brain regions associated with pain and emotion is crucial in shaping self-related experiences in IBS patients (Hall et al., 2010; Mayer et al., 2023), it is more likely that the right CH serves as a crucial structural bridge supporting this interaction in IBS. Therefore, it may be sensitive to acupuncture effects and could be useful in understanding the mechanism of pain treatment.

Interestingly, we found that patients’ perceptions of changes in their QOL were associated with neuroplastic changes in the WM tracts of the right cingulum bundle WM tract. Previous research has revealed that adolescents with IBS showed decreased FA in the right dorsal cingulum bundle compared to controls (Hubbard et al., 2018; Witt et al., 2019). On one hand, abnormalities in FA are associated with anxiety (Ellingson et al., 2013), while on the other hand, decreased FA in the cingulum has been linked to increased pain catastrophizing (Chen et al., 2011). Indeed, there is no significant correlation between HAMA and IBS–SSSs and changes in the cingulum in our findings. However, the correlation of QOL with both HAMA and IBS–SSS suggests that IBS patients’ perception of their QOL depends on their level of pain and anxiety. So, changes in the cingulum might be involved in emotional processing and pain regulation, which are relevant to IBS symptoms (Mayer et al., 2015). The mediation of these factors on neuroplasticity in the right CC in IBS requires further estimation in future studies.

## 5 Limitations

Our study has shown how the WM tracts connected the thalamus and cingulate system after acupuncture treatment. However, several limitations should be considered when evaluating the current findings. First, our longitudinal study design does not allow us to differentiate the brain effects of acupuncture treatment and other factors, such as natural disease course changes or psychological placebo effects. In our study, to minimize the other non-acupuncture treatment factors on the neuroplasticity results, no other treatment was given during the acupuncture treatment, and clinical and psychological measurement data, as well as high-resolution diffusion MRI data, were obtained immediately when each subject came to the hospital for treatment and the day after completing acupuncture stimulation for 6 consecutive weeks. However, some studies reported that although there is clinically meaningful improvement in IBS symptom severity or IBS-related QOL after acupuncture, there are no statistically significant benefits of acupuncture relative to a sham acupuncture control (Manheimer et al., 2012; Qi et al., 2022). This implies that placebo stimulation may also improve IBS symptoms, whether the mechanism behind this is related to an individual’s preference for acupuncture treatment has not yet been determined. However, the lack of a blind control group makes it impossible to directly quantify the effectiveness of acupuncture treatment for IBS and the neuroplasticity of the brain structural characteristics. Future studies will add more experimental control groups (sham acupuncture, drug treatment, and psychotherapy), combine multimodal imaging data, and more comprehensive questionnaires to help specify the neuroplasticity of the brain system of IBS patients of acupuncture. Second, individual patients with other types of IBS were incorrectly included in the study due to uncertainty in patient self-reporting which might have qualitatively differed from those of the IBS-D participants although all patients were indeed diagnosed with Rome III criteria for diagnosing irritable bowel syndrome (Drossman, 2006). Since different types of IBS patients require different treatments, there may also be differences in the neural characters (Wilder-Smith et al., 2004), and it is difficult to predict exactly how this will affect our analysis. In the future, comparative analyses could be performed by expanding the sample size and also including other types of IBS patients to investigate the potential involvement of additional fiber in the same volume. In the current study, we analyzed the WM tracts connected thalamus and cingulate system because we have a strong hypothesis that IBS patients experience long-term digestive abnormalities, pain, and may often suffer from psychological disturbances such as depression and anxiety, with attendant abnormalities in the functional activity and structural properties of the CC, thalamus related network (Weaver et al., 2016; Mayer et al., 2023). However, we cannot exclude the possibility that other WM fibrillar connections may also be associated with IBS, and future studies should consider other connection bundles. Furthermore, only one observation time point after acupuncture treatment was set up in this study, more additional time points will be added to improve the study for thorough observation of acupuncture effect.

## 6 Conclusion

This longitudinal study elucidates the improvements of clinical and psychiatric markers in IBS-D patients following acupuncture treatment, as well as the neuroplastic changes in the brain’s WM fiber tracts related to IBS-D during recovery. We found significant alterations in WM diffusion properties in thalamic and cingulate-related networks in IBS patients. This change was particularly in the right thalamic radiation and right CH bundle. At the same time, we found that changes in WM fiber tracts were associated with improved IBS symptom severity, anxiety levels, and overall perception of QOL in IBS-D patients, separately. Our findings reveal the critical role of the thalamic radiation, cingulate hippocampus, and cingulate gyrus in IBS-D, particularly the WM tracts in the right hemisphere, which can be considered as different potential “biomarkers” of clinical manifestations and mental recovery from IBS-D.

## Data Availability

The original contributions presented in the study are included in the article/supplementary material, further inquiries can be directed to the corresponding authors.

## Footnotes

^1^ www.fmrib.ox.ac.uk/fsl

^2^ https://github.com/jyeatman/AFQ

^3^ https://github.com/vistalab/vistasoft/tree/master/mrDiffusion
